# Simulator-Based Training Sustainably Improves Confidence, Theoretical Knowledge, and Success Rates in Lumbar Puncture Among Medical Students: A Prospective Case–Control Study

**DOI:** 10.1177/23821205261427178

**Published:** 2026-04-19

**Authors:** Jakob Stögbauer, Samira Jonas, Wenlin Hao, Victoria Schegerer, Steffen Kottackal, Laurin Schappe, Sergiu Groppa, Mathias Fousse

**Affiliations:** 1 Department of Neurology, 160536 Saarland University Medical Center, Homburg, Germany; 2 Department of Neurology, 234040 Saarland University, Homburg, Germany

**Keywords:** lumbar puncture, education, simulator, students, undergraduates, tutorial

## Abstract

**Background:**

Lumbar puncture (LP) is an essential tool in everyday clinical neuropsychiatric practice. It has been demonstrated that, among junior doctors, the procedure is associated with elevated levels of stress and anxiety, which can ultimately result in patient suffering. Notwithstanding the evident challenges, innovative teaching methodologies have not yet become a standard in daily clinical practice, despite the existence of different LP simulators.

**Methods:**

The prospective case–control study comprised 20 undergraduate medical students who participated in a tutorial that encompassed both theoretical knowledge and practical training regarding LP, utilizing a simulation model (“tutorial group”). Twenty-one students who were instructed in LP technique according to teaching methods from everyday clinical practice were used as controls (“control group”). A questionnaire was administered to all students, enquiring about confidence levels, theoretical knowledge, and their proficiency when handling the puncture needles. Moreover, the success rate in performing their initial 4 LPs was measured.

**Results:**

The students in the tutorial group demonstrated significantly higher success rates in performing their first 4 LPs (median 3 vs. 1, *p* < 0.001). Furthermore, confidence levels and safety when handling the puncture needles were significantly higher in the tutorial group, as well as knowledge regarding theoretical backgrounds. Higher confidence and safety in needle handling were strongly associated with a higher success rate.

**Conclusion:**

Short simulator-based tutorials have been shown to be an effective method of increasing the skills of young physicians and students in LP, and thus enhancing patient safety. Analogous approaches should be implemented on a large scale in medical education.

## Introduction

Lumbar puncture (LP) is an important diagnostic and therapeutic procedure in clinical practice, used by various medical specialties (including neurology, pediatry, and oncology). It is used to obtain cerebrospinal fluid.^1^ Medical students and young residents have a great deal of respect for technical procedures. This is primarily due to a reluctance to cause harm to patients.^2^ Stress and fear can have a negative impact on patient comfort and safety during first LPs.^3–5^ Despite this evident issue, the bedside teaching principle of “see one, do one, teach one” remains a common practice in everyday clinical settings,^6^ despite the obvious weaknesses of this strategy.^7^

In order to enhance the self-assurance and quality of the procedure among novices, a range of simulator-based training options were evaluated over time,^8–11^ with some of these even using augmented reality.^12^ It is evident that these can contribute to an improvement of the procedure. However, the majority of studies to date have employed pre–post comparisons or subjective assessments by the physicians conducting the studies. When success rates for LP have been measured at all, these have generally only related to the first puncture. To date, randomized case–control studies comparing different teaching strategies in LP are rare.^8,9,12,13^ Furthermore, the focus of these studies has predominantly been on settings with pediatric patients^13^ and young residents,^14^ with limited attention paid to the inclusion of medical students and on settings with pediatric patients.^13^

The objective of this study was to evaluate the sustainability of the effect of a simulator-based tutorium on theoretical knowledge, self-confidence, technical skills, and the success rate of LP by medical students.

## Methods

### Participants and Tutorial

The prospective case–control study included medical students during the practical year (PJ) in the final stage of medical school at the Department of Neurology of the University of Saarland, Homburg (Germany). Students in the control group were taught in accordance with everyday clinical practice. This meant that they performed their first LPs under the guidance of their supervising resident, having previously observed at least 1 LP. There was no further specific teaching in this group. The students in the tutorial group received an additional 45-min tutorial before the start of the PJ, including an explanation of the theoretical principles of LP and practical exercises on an LP simulator (Kyoto Kagaku Lumbar Puncture Simulator II, SkillsMed Deutschland, Nuremberg, Germany). The same resident always performed the tutorial. The simulator was used to practise handling different puncture needles and dealing with different physical conditions (advanced age and obesity) as well as examination conditions (lying or sitting position). All students in the tutorial group carried out the same procedures, completing at least 5 punctures under the various conditions (see above) on the simulator. Both groups of students then carried out a minimum of 4 LPs on different real-world patients with similar characteristics. The success rate of the punctures (successful extraction of cerebrospinal fluid) was documented. After performance of the 4th puncture, all participants in both the control and tutorial groups completed a self-designed questionnaire comprising 4 Likert scales on self-confidence, theoretical knowledge of indications and contraindications, safety in handling the puncture needles, and satisfaction with resident teaching (each rated with 0–5 points, see Supplemental material). Students were randomly assigned to the respective study groups. None of the participants had any experience with needle handling prior to the start of the study, especially in the context of LPs.

**Figure 1.**
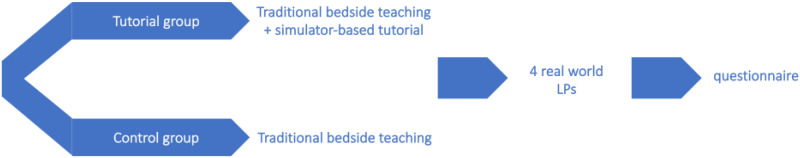
Study design. Abbreviation: LP = lumbar puncture.

The study design is depicted in Figure 1.

### Statistics

Statistical analysis was conducted using SPSS Statistics (version 29.0.2.0). The presence of normal distribution was checked using the Shapiro–Wilk test. Descriptive statistics were described using median and range. Comparison of unpaired, nonparametric data between different groups was carried out using the Mann–Whitney *U* test. Spearman's correlation coefficient was used to analyze bivariate correlations. A statistical analysis was deemed to be significant if the *p*-value was <0.05.

## Results

**Figure 2.**
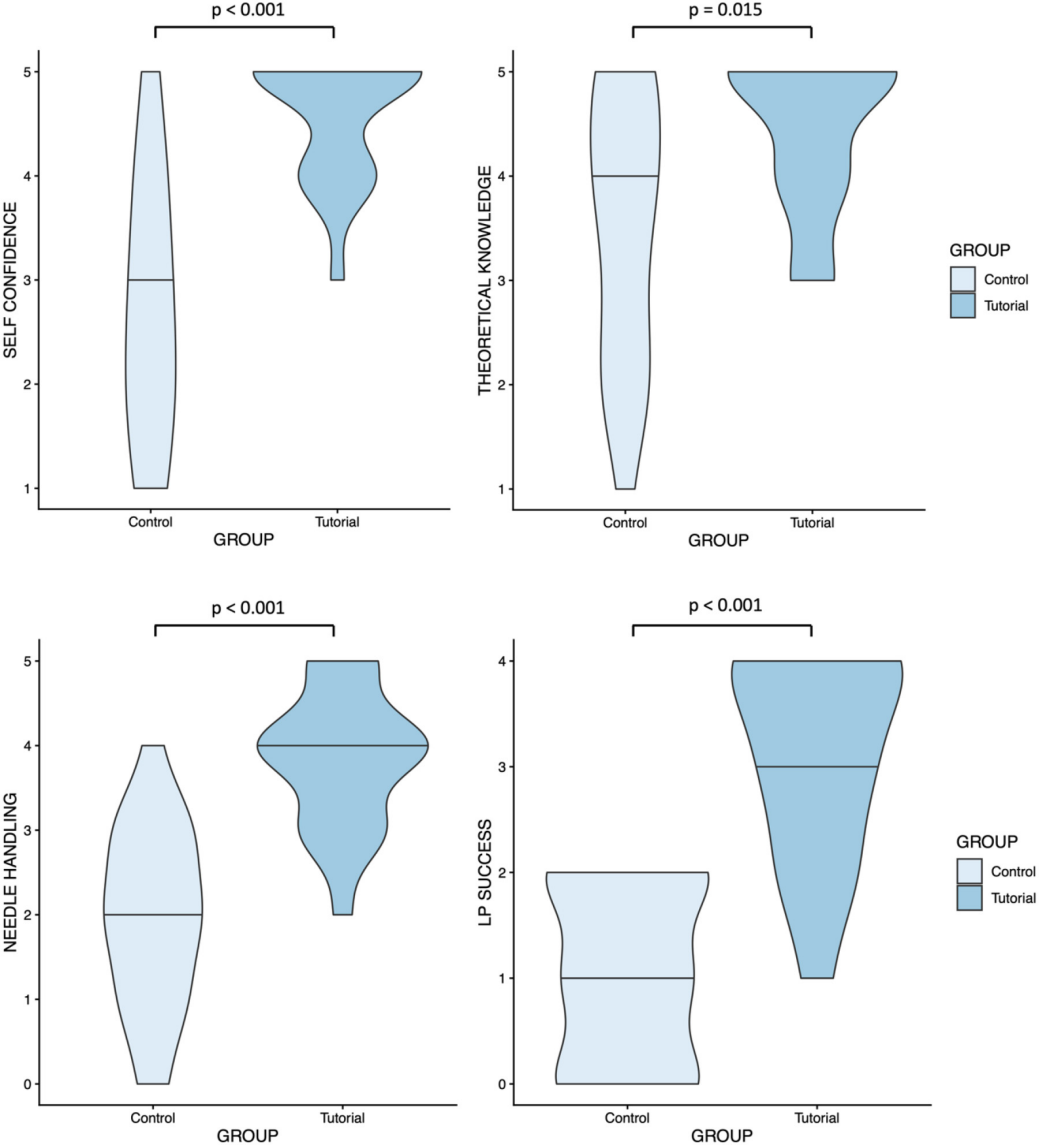
Violin plots showing differences in the students’ evaluation of their preparation for the first lumbar puncture (LP), knowledge of (contra)indications, handling of the puncture needles, and success rate for the first 4 LPs between the control group and the tutorial group.

A total of 41 medical students (20 in the tutorial group and 21 in the control group) participated in the prospective study. At inclusion, all of them were at the beginning of their PJ. The students in the tutorial group demonstrated a significantly higher median success rate in performing their first 4 LPs (3.0 vs. 1.0, *p* < 0.001). It was also observed that the group demonstrated a notably higher level of self-confidence (*p* < 0.001), theoretical knowledge (*p* = 0.015), and safety when handling the puncture needles (*p* < 0.001; see Figure 2). Higher levels of self-confidence (*r* = 0.659, *p* = 0.001) and safety when handling the puncture needles (*r* = 0.706, *p* < 0.001) were found to be significantly associated with the number of successful punctures. There were no significant differences in satisfaction with the care provided by the resident physician.

Results of the questionnaire and success rate are shown in Table 1.

**Table 1.** Results of the Questionnaire and Success Rate in the First 4 Lumbar Punctures (LPs).

	Controls	Tutorial Group	*p*
***N***	21	20	
**Confidence in front of the first LP, median (range)**	3 (1-5)	5 (3-5)	**<0.001**
**Theoretical knowledge, median (range)**	4 (1-5)	5 (3-5))	**0.015**
**Handling LP needles, median (range)**	2 (0-4)	4 (2-5)	**<0.001**
**Satisfaction with the resident, median (range)**	5 (4-5)	5 (4-5)	0.949
**Count of successful punctures, median (range)**	1 (0-2)	3 (1-4)	**<0.001**

Significant correlations (*p *< 0.05) are marked in bold text.

## Conclusions

The simulator-based tutorial led to a higher level of confidence among the students, as well as to a safer handling of the puncture needles and, in particular, to a significantly higher success rate for the first independent LPs. The median of 3 (out of 4) successful punctures indicates that the skills learned can be applied consistently. It was also demonstrated that greater self-confidence and experience in needle handling lead to a higher success rate for LPs. These results are particularly noteworthy, given that students in the control group were also highly satisfied with the care provided by the respective residents. Despite receiving good training, the students performed significantly worse in their first independent LPs. The present study's key strength is its use of a case–control approach and its observation of 4 punctures. Previous research was mostly limited to the first puncture (if success rates were analyzed at all) and to pre/post-comparisons of the same cohort.^9^ When different teaching methods were compared, the simulator-trained group often demonstrated an advantage in LP success rates,^8^ though not always.^12^ Considering the 4 LPs involved, our results are particularly significant in showing the sustainability of the learned skills. The finding that the simulator tutorial was associated with higher self-confidence and better performance is consistent with the results of similar studies.^8–10^ The same applies to the finding that higher self-confidence led to better results.^8,9^ Additionally, it should be noted that our training programme was designed specifically for medical students and not residents (as is usual in previous works). First LPs of these are carried out under the supervision of resident doctors, thus ensuring optimum patient safety. The earlier start of the training programme is likely to facilitate a smoother transition into the residency.

It is recommended that a consistent approach be adopted across the board for the training of medical professionals, with the aim of minimizing the stress experienced by trainees and the risks posed to patients during initial LPs. It would also be conceivable to specifically train for difficult physical conditions.

## Supplemental Material

sj-docx-1-mde-10.1177_23821205261427178 - Supplemental material for Simulator-Based Training Sustainably Improves Confidence, Theoretical Knowledge, and Success Rates in Lumbar Puncture Among Medical Students: A Prospective Case–Control Study Supplemental material, sj-docx-1-mde-10.1177_23821205261427178 for Simulator-Based Training Sustainably Improves Confidence, Theoretical Knowledge, and Success Rates in Lumbar Puncture Among Medical Students: A Prospective Case–Control Study by Jakob Stögbauer, Samira Jonas, Wenlin Hao, Victoria Schegerer, Steffen Kottackal, Laurin Schappe, Sergiu Groppa and Mathias Fousse in Journal of Medical Education and Curricular Development
