# Supplementary material for: Simulator-Based Training Sustainably Improves Confidence, Theoretical Knowledge, and Success Rates in Lumbar Puncture Among Medical Students: A Prospective Case–Control Study
Source: J Med Educ Curric Dev. 2026 Apr 19;13:23821205261427178. doi: 10.1177/23821205261427178 (PMC13103488; doi:10.1177/23821205261427178)
Supplement: sj-docx-1-mde-10.1177_23821205261427178 - Supplemental material for Simulator-Based Training Sustainably Improves Confidence, Theoretical Knowledge, and Success Rates in Lumbar Puncture Among Medical Students: A Prospective Case–Control Study [file sj-docx-1-mde-10.1177_23821205261427178.docx]

**Supplement**

**Supplement Figure 1:** Evaluation form (Likert scale).
